# Three-dimensional morphology of bacterial community developed on the index-matched materials

**DOI:** 10.1038/s41598-021-98943-4

**Published:** 2021-09-30

**Authors:** Chigusa Okano, Kyosuke Takabe, Tomohiro Hirayama, Nobuhiko Nomura, Yutaka Yawata

**Affiliations:** 1 Faculty of Life and Environmental Sciences, University of Tsukuba, Tsukuba, Ibaraki 305-8572 Japan; 2 Graduate School of Life and Environmental Sciences, University of Tsukuba, Tsukuba, Ibaraki 305-8572 Japan; 3 Microbiology Research Center for Sustainability (MiCS), University of Tsukuba, Tsukuba, Ibaraki 305-8572 Japan

**Keywords:** Imaging, Microbiology

## Abstract

Herein, we demonstrate that the use of index-matching materials (IMMs) allows direct visualization of microbial cells maintained at a solid–liquid interface through confocal reflection microscopy (CRM). The refractive index mismatch induces a background reflection at the solid–liquid interface that dwarfs the reflection signals from the cells and results in low-contrast images. We found that the IMMs sufficiently suppressed the background reflection at the solid–liquid interface, facilitating the imaging of microbes at the solid surface using CRM. The use of IMMs allowed quantitative analysis of the morphology of the mesh-like structure of *Pseudomonas aeruginosa* biofilms formed under denitrifying conditions, which led us to propose a novel structural model of the highly porous biofilm structure. These results indicate that the use of CRM coupled with an IMM offers a unique and promising tool for probing the dynamics of biofilm formation, along with visualization of environmental organisms and newly isolated bacteria, for which transformation methods are difficult to establish.

## Introduction

Confocal reflection microscopy (CRM) has been used to visualize the three-dimensional distribution of intact microbiological samples. Distinct from the better-known fluorescence confocal laser scanning microscopy, CRM detects incident light scattered by opaque samples, thereby eliminating the need for epifluorescent tagging^1,2^. Thus, CRM offers a unique solution for analyzing the three-dimensional (3D) structure of microbial communities such as microcolonies, biofilms, and biofouling communities in a tag-free fashion^3,4^.

However, CRM imaging of cells directly adhering to a solid surface is often hindered by the refractive index (RI) mismatch between the solid and liquid phases. Many bacteria exist in a surface-associated aggregate referred to as biofilm rather than in a planktonic state, and bacterial adhesion to the surfaces is a key process for understanding the dynamics of biofilm formation^5,6^. Microscopic studies of biofilm formation typically involve maintaining biofilms on a glass surface for optical access^5–7^. The RI mismatch at the glass-medium interface causes a strong background reflection of the incident light, which dwarfs the reflection signals of the cells. For example, the RIs of the coverslip and culture media, such as Luria–Bertani (LB) medium and M9 minimal medium, are approximately 1.5 and 1.3, respectively^8,9^. The difference between the two RIs is large enough to cause background reflection at the interface; thus, the CRM image at the glass–liquid interface suffers from poor contrast^3^. This technical dilemma, which stems directly from the fact that cells must be maintained on a transparent material to warrant optical access, has kept the CRM from excreting its full potential in tag-free visualization studies of surface-associated microbial communities.

Here, we propose a technical principle to counter this dilemma, where we provide microbes with an optically transparent, yet reflection-free surface to associate and allow biofilms to form. Specifically, we have demonstrated the use of index-matching materials (IMMs) as adhesion surfaces to allow for the direct visualization of microbial cells maintained at a solid–liquid interface using CRM. IMM cancels the RI mismatch at the interface; thus, it has been utilized to control the transmission characteristics in various optical devices and systems^10–12^. We examined the effectiveness of two different types of materials, fluorinated polymers and hydrogels, as IMMs. We overlaid the two IMMs on the glass surface and showed that both materials sufficiently canceled the background reflection at the solid–liquid interface. We demonstrated that, with the aid of IMMs, CRM can be used to visualize the entire biofilm structure, including the parts directly interacting with the substrate surface, without the use of any tags. Furthermore, using CRM coupled with an IMM, we probed the structural details of a biofilm formed under denitrifying conditions, where common fluorescent proteins fail to mature and function. Our quantitative image analysis of the characteristic mesh-like biofilm of *Pseudomonas aeruginosa* formed under denitrifying conditions revealed a unique structural principle, where multiple layers of “2D-mesh” made of elongated cells oriented quasi-horizontally forms lofty and porous 3D microcolonies.

## Results

### Fabrication of IMM layers

**Figure 1:**
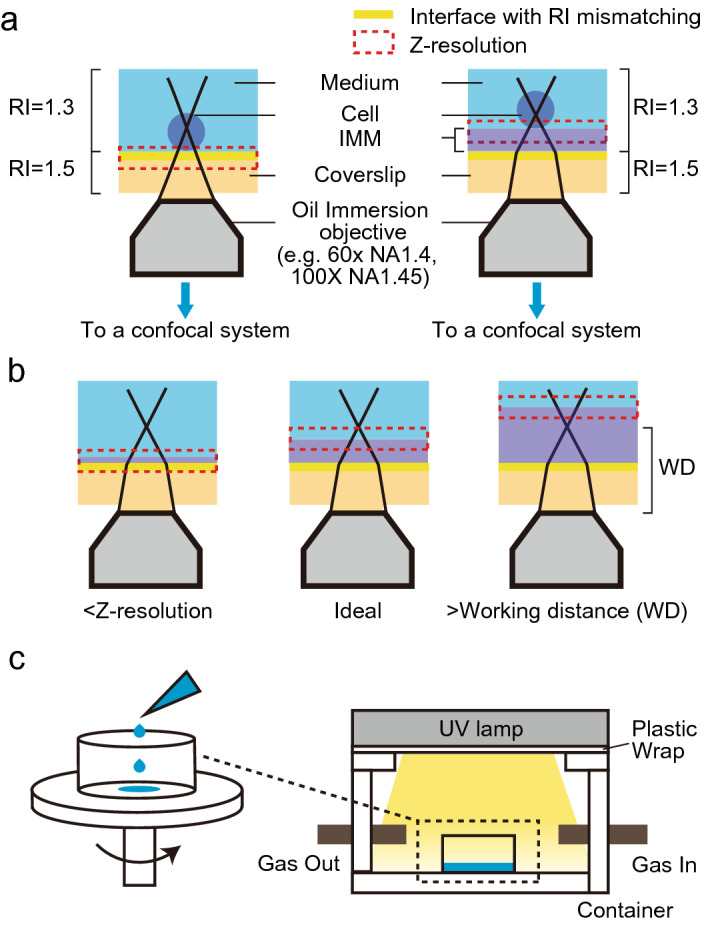
Experimental setup. (**a**) Schematic illustration of experimental setup of conventional type (left) and present type (right). (**b**) Schematic illustration of layer thickness under given z-resolution and working distance. (**c**) The schematic for preparation of fluorinated acrylic polymer layer.

Figure 1a illustrates the schematics of the IMM-implemented experimental setup for observing surface-associated microbes. For effective index matching, the layer thickness should be greater than the z-resolution, such that the optical slice is free of reflection at the interface of the glass and IMM (Fig. 1b). The z-resolution of the confocal system has been defined as the axial full width at half maximum of the point spread function of the confocal system, and it depends on the numerical aperture (NA) of the objective, the wavelength, and the RI of the medium, and the pinhole size^13–15^. For example, for the 60 × NA 1.4 and 20 × NA 0.75 objectives used in this experiment, a minimum layer thickness of 0.5 µm and 2.8 µm is required at the pinhole size of 1.2 airy unit (AU), respectively. Note that the presence of IMM between the sample and glass coverslip should lower the NA of an oil-immersion objective^10^. For example, when the thickness of the IMM is 20 µm, the effective NA of NA = 1.4 oil-immersion objective will be 1.33 (~ 1.7-fold deterioration in z-resolution).

Figure 1c illustrates the schematic for the preparation of a fluorinated acrylic polymer (MY-133-EA, RI = 1.33; My Polymers Ltd.) layer using photocuring. First, the MY-133-EA solution was spin-coated onto glass substrates. The required amount of solution may vary depending on the size of the glass substrate, although we found that spotting 50 µL was sufficient for generating a layer of ~ 20 μm thickness on a coverslip of 35 mm in diameter in a glass bottom dish. The centrifugal force applied to the spin coater is a crucial parameter that determines the layer thickness. For the viscosity of the MY-133-EA solution (2300 cp), we found that spin-coating at 4000 rpm for 30 s resulted in a virtually uniform (standard deviation; SD = 1.9 µm, n = 5) and a reproducible layer, which was 18 μm thick after photocuring (SD of average thickness = 0.25 µm, n = 3). After spin coating, the coated glass coverslip was placed under UV irradiation for photo-initiated curing. The wavelength of 300–400 nm is suitable for photocuring, and we found that with a 365-nm lamp under an atmosphere of nitrogen for 1 min is sufficient for complete curing. The resulting layer should have an elastic modulus of 3.6 MPa (https://www.mypolymers.com/sites/polymers/UserContent/files/MY-133-EA%20161229.pdf) and be free of any residual adhesiveness. We recommend a tactile test for inspection, as the successfully formed layer should feel solid after the photocuring process under the gloved fingertip. After photocuring and inspection, the coated layer was ready for sample loading.

An acrylamide-based hydrogel layer was prepared via chemical gelation. To cast a gel slab, a frame of a silicon gasket was placed on a glass bottom dish. A pre-gel aqueous solution of poly(*N*-hydroxymethyl acrylamide) (8 w/v%) was poured into a frame and covered with a glass coverslip. The top coverslip was removed after incubation for 30 min at room temperature (25 °C). The thickness of gel can be adjusted using a silicone gasket of different thickness, although we recommend > 0.5 mm for the sake of tensile strength and rip resistance. The average thickness of the gel with a 0.5 mm thick gasket was 0.536 mm (SD = 31.8 µm, n = 3). Note that, by its nature, the dimension of the hydrogel can drift with either drying or swelling, depending on the moisture of the storage environment.

An agar gel layer was prepared by physical gelation. A droplet of melted agar solution (0.8 w/v%) was placed on a coverslip and covered with another coverslip, followed by cooling at room temperature (25 °C) for gelation to form a layer. One side of the coverslip was then removed. This process resulted in a layer thickness of ~ 0.5 mm.

### IMMs cancel background reflection

**Figure 2:**
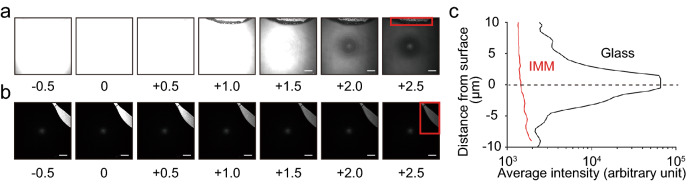
Z-stack images of the interface between (**a**) glass and water or (**b**) MY-133-EA and water. Numbers under images show z-positions from the interface (unit: μm). The red frame shows the region of paint mark. The z-position of the interface was defined as the position where the average signal intensity of the paint mark is maximum. The images were obtained by optical system equipped with a 561-nm continuous wave laser, a 60 × oil-immersion lens (NA = 1.4, RI = 1.515) and a pinhole of 1.2 AU. The z-resolution is 0.5 μm. Bars are 20 μm throughout. Note that deterioration of NA by IMM was not taken into account to calculate resolution. (**c**) Average signal intensities in a field of view around the solid–water interface. The region of the paint mark was excluded from calculation.

The use of an IMM successfully canceled the background reflection at the interface between the water and solid surfaces. Figure 1a shows the placement of the IMM and the experimental setup, where the solid surface with a paint mark was illuminated using a laser, and the reflected light was detected as a signal in an inverted confocal system. The images were obtained using an optical system equipped with a 561-nm continuous wave laser, a 60 × oil-immersion lens (NA = 1.4, RI = 1.515), and a pinhole of 1.2 AU. In this setting, the z-resolution is 0.5 μm. Note that deterioration of NA by IMM was not taken into account to calculate resolution. We measured the level of background signals at the solid–liquid interface, with and without MY-133-EA overlaid on glass as an IMM. Figure 2a,b show the z-stack images near the interface without and with MY-133-EA, respectively. The use of MY-133-EA makes the paint mark clearly visible, whereas it is hardly visible without MY-133-EA owing to the strong background reflection along the z-axis from the interface. Figure 2c shows the average signal intensities of the field of view along the z-axis with and without MY-133-EA. In the absence of MY-133-EA, a strong background signal peak appears at the interface. This signal was almost eliminated by the use of MY-133-EA. These results confirm that the IMMs in this study can counter the background reflection at the solid–liquid interface.

### Cell imaging at the interface between liquid and IMM

**Figure 3:**
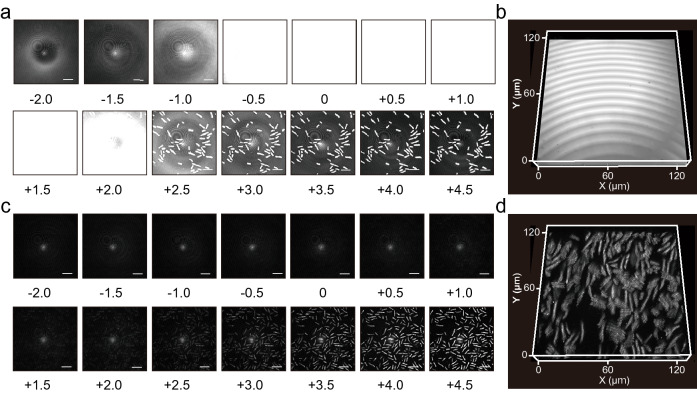
Z-stack images and 3D projection of *S. pombe* JY1 around the (**a**, **b**) glass surface or (**c**, **d**) MY-133-EA surface. Numbers under z-stack images show the z-positions from the interface (unit: μm) and bars are 20 μm throughout. The images were obtained by optical system equipped with a 561-nm continuous wave laser, a 100 × oil-immersion lens (NA = 1.45, RI = 1.515) and a pinhole of 1.2 AU. The z-resolution is 0.5 μm. Note that deterioration of NA by IMM was not taken into account to calculate resolution. The 3D projection depicts the range of 20.5 μm along vertical z-axis.

**Figure 4:**
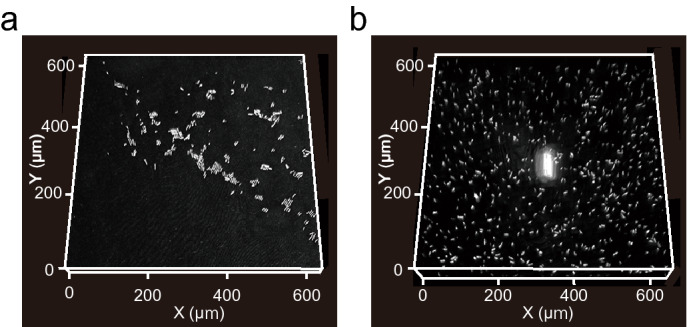
The 3D projection of *S. pombe* JY1 on (**a**) agar gel or (**b**) poly(*N*-hydroxymethyl acrylamide) hydrogel surface. The images were obtained by optical system equipped with a 561-nm continuous wave laser, a 20 × dry lens (NA = 0.75, RI = 1.0) and a pinhole of 1.2 AU. The z-resolution is 2.8 μm. The projection of (**a**, **b**) depicts the range of 33 and 102.5 μm of height along the vertical z-axis, respectively.

IMMs allowed clear visualization of *Schizosaccharomyces pombe* cells resting directly on glass using CRM (Fig. 3). We obtained z-stack images of cells on the glass or on the IMM and represented them as 3D projections of the cells. The images were obtained using an optical system equipped with a 561-nm continuous wave laser, a 100 × oil-immersion lens (NA = 1.45, RI = 1.515), and a pinhole of 1.2 AU. In this setting, the z-resolution was 0.5 μm. Note that deterioration of NA by IMM was not taken into account to calculate resolution. The cells on the glass were hardly imaged in the range of − 0.5 to 2.0 μm, along the z-axis, from the interface (Fig. 3a), and the resulting 3D projections were far from the actual cell morphologies (Fig. 3b). In contrast, cells were successfully imaged without background reflection using MY-133-EA (Fig. 3c); thus, the resulting 3D projections clearly resembled cell morphologies (Fig. 3d). The average signal intensity for the background (the regions other than cell regions, n = 20) of the coated coverslip was 86.2 (arbitrary unit), while that for the coverslip was 3450. Meanwhile, the average of the baseline signal intensity without anything on the stage was 86.4, which suggested that the residual background of the coated surface was negligible. Note that we selected a z-slice of + 1 µm from the surface for these calculations because the intensity of the glass surface was saturated. The signal-to-background ratio at the same z-slices as described above was 1.8 and 15.8 without and with MY-133-EA, respectively; therefore, MY-133-EA allowed roughly 8.8-fold improvement in signal-to-background ratio with yeast sample. The 3D projections of cells were also achieved using other IMMs, namely an agar hydrogel and an acrylamide-based hydrogel (Fig. 4), that are the same types of gels routinely used for gel electrophoresis and plate cultures^16^. These results indicate that IMMs overlaid on the glass presented a lower background reflection, resulting in a better visualization of the cells with CRM.

### Temporal monitoring of initial biofilm formation process

**Figure 5:**
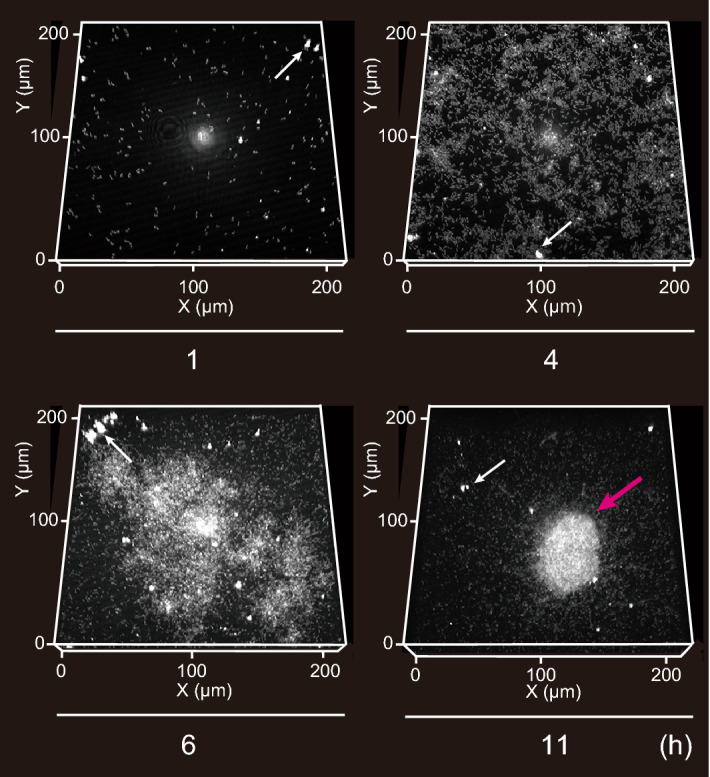
The 3D projection of the initial biofilm formation of *P*. *aeruginosa* PAO1 on the MY-133-EA layer at 1, 4, 6, and 11 h. The images were obtained by optical system equipped with a 561-nm continuous wave laser, a 60 × oil-immersion lens (NA = 1.4, RI = 1.515) and a pinhole of 1.2 airy unit. The z-resolution is 0.5 μm. Note that deterioration of NA by IMM was not taken into account to calculate resolution. Red arrow indicates a microcolony (Height = 25 μm). White arrows indicate debris. The 3D projection of 1, 4, 6 and 11 h, illustrates the range of 0 μm (i.e. surface) to 11, 11, 21, 41 μm of height along the vertical z-axis, respectively.

We temporally monitored the initial biofilm formation on IMM using CRM. We obtained z-stack images of the initial biofilms on MY-133-EA at predetermined time points up to 11 h and reconstructed the images into 3D projections of biofilm formation (Fig. 5). The 3D projections successfully represented the initial biofilm formation process, where cells adhered to the solid phase, grew, and formed further microcolonies (a microcolony is indicated with a red arrow), demonstrating that the use of an IMM allows visualization of biofilm development in a tag-free manner and that the fluorinated polymer layer supports bacterial growth and biofilm formation.

### Structural analysis of bacterial biofilms formed under denitrifying conditions

**Figure 6:**
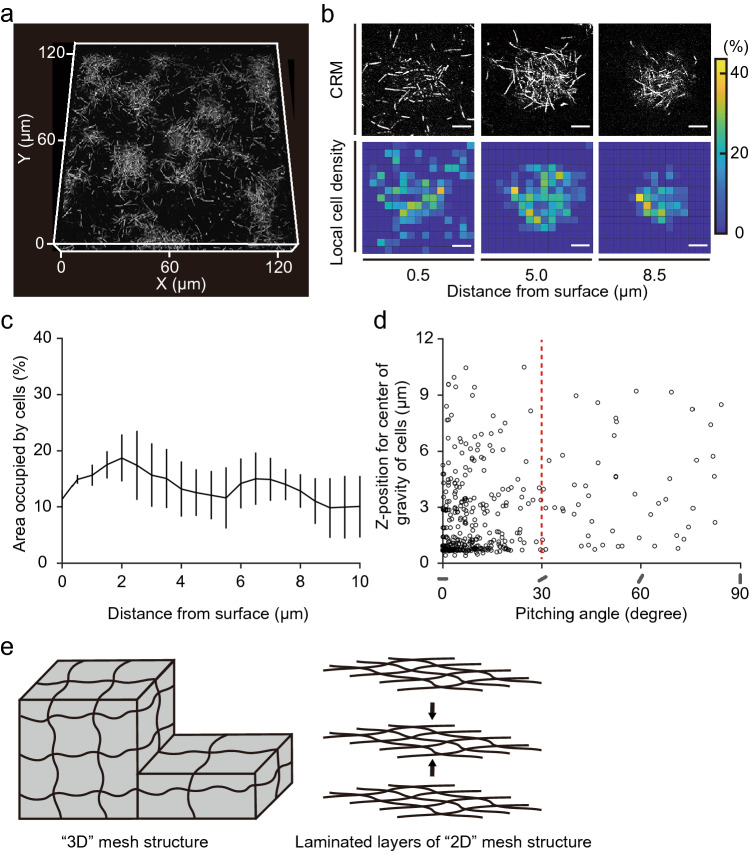
Structural characteristics of *P. aeruginosa* PAO1 WT biofilms formed on the MY-133-EA layer under denitrifying conditions. (**a**) The 3D projection of the biofilms grown under denitrifying condition for 22 h. The images were obtained by optical system equipped with a 561-nm continuous wave laser, a 100 × oil-immersion lens (NA = 1.45, RI = 1.515) and a pinhole of 0.7 AU. The z-resolution is 0.4 μm. Note that deterioration of NA by IMM was not taken into account to calculate resolution. The projection depicts the range of 17.5 μm along the vertical z-axis. (**b**) Optical slice images within microcolony (upper panels) and local cell density presented as a heatmap (lower panels). Numbers below the panels denote the distance from the surface. Bars are 6 μm and the matrix size for the heat maps is 2.4 μm × 2.4 μm. (**c**) Average area occupation rate by cells in the representative microcolony (n = 3). The x-axis denotes the distance from the surface. Error bars denote SDs. (**d**) Pitching angles of individuals cells (○) in the microcolonies relative to the horizontal plane. The y-axis denotes the z-position for the center of gravity of cells, where zero indicates the surface. (**e**) Schematic illustration of the “3D mesh” structure (left) and laminated layers of the “2D mesh” structure (right).

The fluorescent protein tagging technique has fostered an understanding of the structural characteristics of bacterial biofilms^17–20^. However, anaerobic conditions hinder the maturation of commonly used fluorescent proteins^21–23^. Therefore, to date, quantitative information on biofilm structures under anaerobic conditions has been limited. Here, using IMMs, we enumerated the structural characteristics of bacterial biofilms under denitrifying conditions by CRM in a tag-free fashion. The *P. aeruginosa* PAO1 WT biofilms grown on the MY-133-EA layer for 22 h exhibited a characteristic mesh-like structure composed of elongated (i.e., filamentous) cells (Fig. 6a,b), in agreement with previous reports^24,25^. The CRM provided a clear z-stack image of the entire biofilm structure formed on the MY-133-EA layer, from cells directly adhering to the surface to the summit of the microcolonies, which allowed us to analyze the distribution and orientation of each individual cell in the biofilms. The analysis of average local cell density (area occupation rate by cells, n = 3) revealed that, inside the mesh-like structure, local cell density was considerably low and remained almost constant through the z-position (Fig. 6c). The cell occupied 10.0%, 13.4% and 15.8% at the top, mid-height, and bottom of the microcolony structure, respectively. This result implies that the mesh-like structure allows biofilm growth while maintaining a uniformly porous structure, which is supposedly advantageous in material flux^25^. Next, we analyzed the pitching angles of individual cells relative to the IMM surface within the microcolonies. Our initial expectation was that the cells would be randomly oriented, as previously reported with a short bacillus *Vibrio cholerae*^26^ under aerobic condition, thus forming a “3D” mesh, similar to the structure of a polymer matrix. Surprisingly, our analysis revealed that the pitching angle between the long axis of an elongated cell and the horizontal plane rarely exceeded 45°, and the majority (87%) of cells exhibited an angle less than 30° (Fig. 6d, population average = 13.4°). This result suggests that the characteristic pore-rich, mesh-like microcolonies consist of laminated layers of loose “2D” mesh (Fig. 6e), which is “knitted” by nearly horizontal filaments of elongated cells, rather than a “3D” mesh structure that requires members that span vertically.

## Discussion

In the present study, using an IMM as a reflection-free optical window/adhesion surface, we visualized the 3D structure of bacterial biofilms formed under denitrifying conditions in a tag-free fashion. Quantitative image analysis revealed the structural details of the characteristic mesh-like microcolony structure formed under denitrifying conditions, including a remarkable lack of variation in cell orientation and the resulting quasi-uniformly porous structure.

Our results confirmed that, in the experimental setup suitable for biofilm study, the IMM eliminated the RI mismatch between the liquid and solid phases, and the IMMs sufficiently suppressed background reflection at the liquid–solid interface (Fig. 2). Meanwhile, quarter-wavelength anti-reflection coating, often referred to as AR coating, is another well-known method for controlling reflection at the interface. Compared to AR coatings, IMMs have several unique characteristics. First, while the effect of the AR coating is wavelength-specific, the effect of an IMM is independent of the wavelength. This is due to the fact that the thickness of AR coating needs to be close to a quarter of the wavelength of the incident light. While the fabrication of a broad range of AR coatings is possible, it requires a rather complex process such as crafting nanostructures such as moth eye structure and graded structure^27,28^. Second, IMMs are more accessible for in-house fabrication than AR coatings, which generally require vapor deposition or etching. Hydrogel-based variations require materials and instruments that are commonly found in microbiology laboratories.

We have demonstrated the effectiveness of three IMMs, namely fluorinated polymer, an acrylamide-based hydrogel, and agar hydrogel (Figs. 3 and 4). Each material has unique characteristics and advantages (e.g., hydrophobicity, adjustability of RI, store lifetime, and durability) that should best cater to different settings. These characteristics are summarized in Table 1. While fluorinated polymers can be formed with a layer thickness of 10–20 µm, the hydrogel tends to be 0.5 mm thick. This limits the use of hydrogels with high-NA objectives, the working distance of which is typically < 200 µm. Hydrogels are typically hydrophilic, while fluorinated polymers are hydrophobic^29^, although the surface properties of both variations can be tuned by the introduction of hydrophobic or hydrophilic moieties^30,31^. Should the need to adjust the RI of an IMM arise, the hydrogels offer a simpler solution. The RIs of high-water-content hydrogels are in the range of 1.33–1.35^32^ with water as the solvent, while their RI depends on the solvent. Thus, the RI of hydrogels can be tuned simply by using a solvent with different RIs in the fabrication of the hydrogel. While the RI of fluorinated polymers can also be tuned (fluorination of functional groups results in a lower RI^28^), the process involves organic synthesis. Hydrogels are also advantageous in terms of accessibility. While forming a layer of fluorinated polymer requires a spin-coater and UV irradiation instruments, hydrogels only require equipment and materials commonly found in microbiological laboratories that practice electrophoresis. In contrast, fluorinated polymers excel in ease of handling and shelf life. Fluorinated polymers can be stored in dry conditions, whereas hydrogels require a moisturizing environment for storage. Hydrogels also tend to swell under such storage conditions, which could be a problem for a situation that demands exact thickness for the IMM layer. We also found that fluorinated polymers are more durable than hydrogels that can rip, although acrylamide-based hydrogels are more rip-resistant than agar gels due to chemical gelation^33^.

**Table 1:** Characteristics of IMMs.

IMM materials	Thickness	RI adjustability	Hydrophobicity	Storage
Fluorinated polymers	Thin (10–20 μm)	Requires organic synthesis	Typically hydrophobic	Dry
Hydrogels	Thick (0.5 mm)	Adjustable with solvent	Typically hydrophilic	Moisturized environment

The IMM dose sets a few unique constraints on the experimental setup. First, the presence of IMM can take up a fraction of the objective’s working distance. The working distance of high-magnification, high-NA objectives are typically 100–200 µm, while fluorinated polymers can be formed with a layer thickness of 10–20 µm. Second, as discussed in the results section, the presence of IMM should lower the NA of the oil-immersion objective. The use of a water-immersion objective should address this problem. Third, the elastic nature of hydrogel-based IMMs requires a multi-layer structure, for example, a thin hydrogel overlaid on a supporting structure, such as a glass coverslip. The fabrication of thin and rigid IMM slabs should be explored to eliminate the need for a support structure and further streamline the experimental setup. Notably, the contamination of the hydrogel or polymer solution with dust can result in bright debris in the CRM image (Fig. 5, white arrows). Thus, we recommend preparing IMM in a dust-free environment.

In this study, we successfully imaged microbial samples on a solid surface (Figs. 3, 4 and 6) and temporally imaged the initial biofilm formation process (Fig. 5). Furthermore, our quantitative imaging analysis has shed new light on the structure of and cell order inside the characteristic mesh-like microcolony of *P. aeruginosa* formed under denitrifying conditions, where we found the laminated layers of “2D-mesh” knitted with elongated cells that were oriented quasi-horizontally, forming a lofty and pore-rich 3D structure. In contrast, a previous study with *V. cholerae*, which has a short rod-shaped cell morphology (approximately 2 μm), showed that cells are randomly oriented in a growing microcolony^26^. From a biophysical viewpoint, it is intriguing to ask why the pitching angle of the elongated cells can be tightly constrained around being horizontal (Fig. 6d). Differences in cell morphology, especially in cell length (3–10 μm vs. 2 μm), might play a key role in this stark contrast in the distribution of the pitching angle. Elucidation of the exact mechanism and development process for this orderly cell placement, however, would require a detailed time-course study. On the other hand, from an ecological perspective, the porous, mesh-like structure supposedly helps material transport, as suggested previously^25^.

Visualization studies of biofilm structure with confocal microscopy coupled with fluorescent protein reporter tagging technique revealed elaborate 3D microstructures of biofilms, represented by the mushroom-like structure, fostering a body of research on their direct and ultimate ecological factors^18,19^. Such studies, however, mostly focused on biofilms of established laboratory model organisms, often under moderate laboratory conditions. Meanwhile, metagenomic studies suggest that a plethora of indigenous bacteria harbor genetic potential for biofilm formation^34^, and field studies have discovered an example of biofilm formation under extreme conditions^35,36^. In this study, we demonstrated that CRM coupled with IMMs enables the quantitative visualization and analysis of biofilms at single-cell resolution in a tag-free fashion. The technological principle described here then presents a powerful tool to explore the broader spectrum of biofilm structures formed by microbes, for which transformation methods are difficult to establish or apply.

## Methods

### Synthesis of IMM layer

We synthesized a layer of each IMM onto glass substrates, including a glass slip (RI = 1.5255; Matsunami Glass Ind., Ltd., Japan) and a glass bottom dish (AGC Techno Glass Co., Ltd., Japan). An MY-133-EA (MY Polymers Ltd., Israel) layer was prepared by photocuring, wherein we spin-coated the MY-133-EA solution on the glass substrates at 4000 rpm for 30 s (MS-B100; Mikasa Co., Japan), followed by photo-initiated curing by irradiation with UV light from a height of ~ 2 cm using a 365-nm lamp (LUV-16; AS ONE CO., Japan, 1820 µW/cm^2^ at a distance of 5 cm from the source), for 1 min under a nitrogen atmosphere to generate a layer of ~ 20 μm thickness. Before biofilm culture, the coated glass bottom dish was washed with 70% ethanol and then UV-irradiated on a clean bench for 10 min. The layer thickness was measured with a digital micrometer (Shinwa Rules Co., Ltd., Japan). Poly(*N*-hydroxymethyl acrylamide) hydrogels were prepared by chemical gelation^32^. A pre-gel aqueous solution consisting of 8 w/v% *N*-hydroxymethyl acrylamide (Tokyo Chemical Industry Co., Ltd., Japan), 0.2 w/v% *N*,*N*′-methylenebisacrylamide (Tokyo Chemical Industry Co., Ltd., Japan), 0.1 v/v% *N*,*N*,*N*′,*N*′-tetramethylethylenediamine (Tokyo Chemical Industry Co., Ltd., Japan), 0.05 w/v% ammonium peroxodisulfate (Tokyo Chemical Industry Co., Ltd., Japan), and distilled water was poured into a silicon frame of 0.5 mm-thick (SR-50; TIGERS POLYMER Co., Japan) adhered to a glass bottom dish. The frame was sealed with a coverslip and incubated at room temperature (25 °C) for gelation. Agar gels were prepared by physical gelation^32^. The agar (0.8 w/v%, Nacalai Tesque, Inc., Japan) was dissolved in phosphate-buffered saline (PBS; Fujifilm Wako Pure Chemical Co., Japan) while heating. A droplet of agar solution (150 μL) was placed on a coverslip and covered with another coverslip, followed by cooling at room temperature for gelation to form a layer with a thickness of ~ 0.5-mm thick. One side of the coverslip was gently slid off.

### Strains and culture conditions

The strains used in this study were *S. pombe* JY1 and *P. aeruginosa* PAO1^37,38^. *S. pombe* JY1 was cultured in yeast extract-peptone-dextrose (YPD) medium (BD Bioscience, USA) with shaking (190 rpm) overnight at 30 °C. The cells were resuspended in PBS. *P. aeruginosa* PAO1 was cultured in LB medium (Nacalai Tesque, Inc., Japan) while shaking (190 rpm) overnight at 37 °C. For biofilm formation, the culture of *P. aeruginosa* PAO1 was inoculated into fresh LB medium supplemented with 100 mM KNO_3_ (Fujifilm Wako Pure Chemical Co., Japan) to adjust the optical density of the medium at 600 nm of 0.01 and was placed in a 25-μL frame-seal™ incubation chamber (Bio-Rad Laboratories, Inc., USA) to adhere to the MY-133-EA layer. The chamber was sealed with silicon resin and incubated at 37 °C under aerobic conditions. For biofilm culture under denitrifying conditions, cultures were maintained in an AnaeroPack system (Mitsubishi Gas Chemical America, Inc., USA).

### Experimental setup and image analysis

The experimental setup is shown in Fig. 1a. The sample was illuminated with a 561-nm continuous wave laser. The reflected light passed through a half mirror and pinhole was detected with a photomultiplier tube in an inverted confocal system (Nikon A1; Nikon Solutions Co., Ltd., Japan). The average signal intensities in the field of view, except for the region of the paint mark, were calculated by processing the image using a custom MATLAB (MathWorks, Inc., USA) routine. We reconstructed 3D projections from z-stack images using the imaging software NIS-element AR (https://www.microscope.healthcare.nikon.com/products/software/nis-elements/nis-elements-advanced-research, version number: 5.21.00, NIKON Solutions Co., Ltd.). Single cells in the z-stack images were recognized using a custom MATLAB routine, and the cell orientation was calculated using the *regionprops3* function in MATLAB. Cell density inside the microcolony was defined as the percentage of area occupied by the recognized cell regions within an arbitrarily defined microcolony region on each z-slice. The signal-to-background ratio was defined as the ratio of the average intensity of the reflection from the cell regions (n = 20) and that of the background regions (area other than cell regions, n = 20).

## Data Availability

The data generated and analyzed during the current study will be made available from the corresponding author upon reasonable request.
